# Parent-Adolescent Conflict and Its Resolution in Monogamous and Polygamous Bedouin Arab Families in Southern Israel

**DOI:** 10.1100/tsw.2003.99

**Published:** 2003-12-05

**Authors:** Salman Elbedour, Joel M. Hektner, Mohammed Morad, Soleman H. Abu-Bader

**Affiliations:** Department of Human Development and Psychoeducational Studies, School of Education, Howard University, Washington, DC, USA

**Keywords:** polygamy, conflict resolution, public health, Bedouin, Israel

## Abstract

The purpose of this study was twofold: (1) to compare whether children from polygamous family structures significantly differ from children from monogamous family structures with regard to the frequency of parent-child conflict, and (2) whether children from these two structures employ different patterns of family conflict resolution.To address these questions, a random sample of 212 high school students (60.8% monogamous) completed a self-administered survey. The results of MANOVA show no significant differences (p > 0.05) between these two structures with regard to the frequency of parent-child conflict. The results also show similar conflict management styles between these two family structures within each of the following five domains (privacy, school and career, money spending, going out and leisure, and physical appearance).This study is unique in that it is the first empirical research to be conducted in the field of conflict resolution among youth and adolescents in polygamous marital structures and therefore, further investigation is needed to replicate these results utilizing different cross-cultural populations practicing polygamy.

### INTRODUCTION

The unfavorable effects of polygamous marital structures on the well being of children have been established by a wide range of sources[1,2,3,4,5,6,7,8,9]. Compared to their counterparts in monogamous families, children raised in polygamous families commonly exhibit developmental dysfunction, as reflected in many outcomes including poor school performance[10,11], poor mental health status[3,12,13], low self-esteem[14], poor social adjustment[11], and rivalry between full- and half-siblings[15]. Elbedour et al.[16] assign these adverse outcomes not directly to the polygamous marital structure, but to the family dynamics within these polygamous families. Elbedour et al.[16] found support for this proposition not only from research related to polygamous marital structures, but relevant work conducted on children of marital conflict[17,18] in Western societies.

Specifically, after an extensive review of the literature[16] these problems were attributed not to the polygamous family structure itself, but rather to family factors such as competition among wives[19], parental conflict over the father's absences, rivalry between full- and half-siblings[15], uneven treatment of wives by their husband[20], and the child's perception of neglect and abandonment by the father[21]. Other challenges for polygamous families include the financial burden of supporting a larger family and the relative psychological absence of the father[16]. These stressors lead to distress, disagreement, and marital tension, which challenge the parents' ability to care for their children. When a family breaks down in this way, the children bear the greatest burden and often become the target of their parents' frustrations[22,23]. Support for this proposition is also provided by the "spillover" hypothesis[24,25], which holds that the distress, hostility, and preoccupation that arises from marital difficulties is transferred into parenting behavior, resulting in dysfunctional parenting and impaired outcomes for the children. Spousal tension can disrupt parenting[26] and cause mothers to withdraw and become hostile toward their children, and the children themselves may be pressured to take sides in the conflict between the parents[17].

In a recent study by Krishnakumar and Buehler[27], half the parents reported that spousal conflict was a precipitator of parent-child tension. A 1977 study found that the dysfunctional behavior of husbands toward their wives was directly correlated with the dysfunctional behavior of the wives toward their 5-month-old children. A mother's negative assessment of her relationship with her spouse was correlated with negative interactions with her children and between the older siblings[28]. Elbedour et al.[16] pointed out that these family processes account for much of the link between polygamous families and the maladjustment of children.

The present study attempts to explore parent-adolescent conflict and its resolution in monogamous and polygamous Bedouin-Arab Families in Israel. Most of the research that has found more parent-child conflict in polygamous families has been influenced by a Western cultural bias against polygamy, and empirical evidence is lacking to refute or validate this perspective. The current study will provide the data needed to evaluate the accuracy of the previous research. It will compare patterns of parent-child conflict resolution in polygamous families with those in monogamous families. We will assess parent-child conflict resolution styles by examining nine styles of conflict resolution (integrative, avoidance, compromise, accommodating, deception, competitive, mediation, threat against others, and threat against oneself) in five domains (privacy, career orientation, physical appearance, monetary issues, and leisure pursuits). In each domain, we will examine four questions, comparing the responses for monogamous and polygamous families on each one:

1. What styles of conflict resolution are used the most and the least?
2. Who (mother, father, or adolescent) is most influential in the decision-making process in this domain?
3. Who (mother, father, or adolescent) has the right to decide in this domain?
4. How satisfied is the adolescent with the decisions made in this domain?

Compared to their peers in monogamous families, we expect that adolescents in polygamous families will experience a higher rate of parent-child conflict; we also expect them to use different patterns of conflict resolution in the five domains.

Furthermore, because of their increased exposure to marital conflict, we predict that the children in polygamous families will adopt more violent behavior as a means of problem solving. The expectation is partially supported by previous research on marital conflict, conducted in monogamous Western families, which shows that children tend to mimic their parents' conflict resolution styles. A child who is exposed to stress and violence in the family setting will tend to express more hostility and use more violent coping methods[29,30]. A chronic pattern of family violence typically leads to elevated levels of anger, aggression, and violence in the children[31].

Before we proceed to test these hypothesis, it is worth noting that some researchers challenge the fundamental concept that polygamy has a deleterious effect on children, contending that despite the multiplicity of stressors in the polygamous family unit, it does not have a negative impact on children[1,13,15,32]. These authors contend that a polygamous family structure provides benefits for children, including more role models for socialization, more opportunities for receiving attention and affection, and a more secure psychological basis for dealing with stress[12,33]. These authors point out that, far from having a negative effect, in many parts of the world polygamy is practiced by all social groups and "is an expression of a way of life which is deeply embedded in . . . religious and cultural obligation"[34]. For example, in Africa polygamy is considered to be the "most distinctive feature of an African marriage"[35].

Thus, it is likely that the prevalence of parent-child conflict in polygamous families may vary as a function of the surrounding culture and its values. Culture affects children's cognitive processes and has been found to alter the correlation between family variables and child development outcomes[17,36]. It appears that the way children assess and respond to interfamily conflict depends, at least to some degree, on the values of their culture. In addition, Jouriles et al.[37] found that children have the ability to differentiate between conflict that is child-related and other types of family conflict. In sum, the development of children within a polygamous marital structure may best be described as a culturally bound phenomenon, and we must take cultural values into consideration, for they may alter the direction of our hypothesis.

### METHODS

#### Participants

There were 212 participants, of whom 60 (28.3%) were male and 152 were female. About half (54.8%) of the participants were in grade 11, 41.4% were in grade 10, and 3.8% were in grade 12. The sample was drawn from four of the six Bedouin-Arab schools in a Negev Bedouin community in the 2000/2001 school year. The sample in each school was random and subjects completed the scale with the idea that the study needed to assess the type, nature, frequency, and styles of conflicts between children and their parents. Subjects were not informed that the study intended to compare the frequency and styles of parent-child conflicts within polygamous and monogamous families. After completing the scale, subjects were instructed to complete a socio-demographic questionnaire that included items such as age, gender, family type, parental education, and occupation.

The socioeconomic status of the participants' parents was low, as indicated by father's education (58.2% did not finish high school; 8.2% went beyond high school), and occupation (53.0% unemployed; 22.3% in unskilled labor). Mothers' levels of education and occupation were even lower. According to participants' reports of their parents' marital structure, 60.8 % were monogamous, and the rest came from families in which the fathers had two wives.

#### Measures

The parent-child conflict management scale was designed by the first author to assess adolescent conflict management styles in conflicts with their parents. The questionnaire used in the current study was adapted from the Conflict Management Inventory (CMI)[38]. The original CMI assessed five conflict management styles: compromising, avoiding, integrating, accommodating, and competing.

However, unlike the original CMI, which asks generally about the global conflict management style of the subject, the questionnaire used in the current study assessed conflict management styles in each of five specific domains: privacy, career, money, physical appearance, and leisure times (see Appendix). We believe that conflicts between parents and their children tend to be more domain specific in nature than global. A second modification was the addition of more conflict management styles to the assessment. In addition to the five styles included in the original CMI, we added questions to assess four other styles of conflict management: deception, mediation, threat against parents, and threat against the self. Thus, there were nine conflict management styles assessed in each of five domains. Each style within each domain was assessed by one question, rated on a five-point scale from "Definitely not true of me" to "Clearly true in my case." For example, the item on the compromise style within the money domain was "When you have arguments with your parents about the money issue you specified, you use 'give and take' so that a compromise can be made." Styles tended to cohere across domains, as indicated by internal consistency estimates when all five items assessing the same style across the five domains were tested as a scale. Cohen's alpha ranged from 0.60 to 0.80 for seven of the styles, but dropped to 0.54 for mediation and 0.38 for deception.

In addition to the conflict management styles, the questionnaire also asked about a number of other conflict parameters within each domain, such as the frequency of conflict, who wins, who decides, how influential each party was, and how satisfied the adolescent was with the resolution. Finally, information on several demographic variables (parent education, marital status, participant's age, grade, and sex) was also collected.

### RESULTS

The monogamous and polygamous groups did not differ significantly with respect to gender, grade, age, or father's level of education. Table 1 displays the data for these comparisons. To test whether these groups differed on each of the continuous conflict parameters (variables other than the styles of conflict resolution), a MANOVA was conducted for each conflict parameter across the five domains. For example, the five frequencies of conflict variables, one for each domain, were entered into the MANOVA as the set of dependent variables. The dichotomous family structure variable was the independent variable. No significant differences emerged between the two groups on frequency of conflict, $F(5, 163) = 1.30$, $p = 0.268$. Table 2 displays the group means on all of the conflict parameters across all domains. There were also no group differences on the strength of the father's influence on conflict resolution, $F(5, 162) = 1.09$, $p = 0.366$, or the strength of the mother's influence, $F(5, 160) = 0.72$, $p = 0.613$, or the strength of the student's influence, $F(5, 157) = 0.65$, $p = 0.666$. Students were also asked how often they "win" arguments in each domain and how satisfied they were with the outcomes of those arguments. A MANOVA on each of these sets revealed no significant group differences on either variable, $F(5, 169) = 0.41$, $p = 0.839$ and $F(5, 173) = 0.78$, $p = 0.563$, respectively. To test whether there were differences between groups in the proportions of students affirming that they (versus their parents or both) should have the right to decide matters in each domain, chi-square tests were performed on each of these variables. The results from these tests are shown in Table 3, where it can be seen that there were no significant differences on any of these variables.

**TABLE 1**
**Demographic Characteristics of Each Group**

| Characteristic | Monogamous | Polygamous | *p* Value |
|---|---|---|---|
| % Male | 27.9% | 29.6% | 0.788 |
| Father's education | | | 0.908 |
| < Elementary | 39.3% | 40.8% | |
| < High school | 17.9% | 19.7% | |
| High school graduate | 33.3% | 32.9% | |
| > High school | 9.4% | 6.6% | |
| Mean age | 16.4 | 16.4 | 0.716 |
| Mean grade | 10.6 | 10.6 | 0.682 |

*Note:* Chi-square tests were conducted on gender and father's education. t-Tests were conducted on age and grade.

**TABLE 2**
**Group Means in Each Domain on Conflict Parameters**

| Conflict Parameter | Appearance | Career | Money | Going Out | Privacy |
|---|---|---|---|---|---|
| **Frequency** | | | | | |
| Monogamous | 4.21 | 3.99 | 2.72 | 4.55 | 3.96 |
| Polygamous | 3.12 | 3.51 | 3.08 | 4.71 | 4.00 |
| **Father Influence** | | | | | |
| Monogamous | 3.24 | 3.42 | 3.94 | 3.69 | 3.07 |
| Polygamous | 2.81 | 3.67 | 3.77 | 3.67 | 3.11 |
| **Mother Influence** | | | | | |
| Monogamous | 2.95 | 2.87 | 3.03 | 3.09 | 2.70 |
| Polygamous | 3.08 | 3.21 | 2.95 | 3.26 | 3.08 |
| **Participant's Influence** | | | | | |
| Monogamous | 3.78 | 3.98 | 3.06 | 3.53 | 4.05 |
| Polygamous | 3.76 | 3.97 | 3.00 | 3.45 | 3.69 |
| **Frequency participant wins** | | | | | |
| Monogamous | 3.09 | 3.00 | 3.28 | 2.95 | 3.10 |
| Polygamous | 3.09 | 3.03 | 3.08 | 3.05 | 3.23 |
| **Satisfaction** | | | | | |
| Monogamous | 3.33 | 3.50 | 3.56 | 3.33 | 3.26 |
| Polygamous | 3.26 | 3.59 | 3.42 | 2.94 | 3.18 |

Figs. 1 and 2 show the mean score on each conflict resolution style within each domain for monogamous and polygamous families, respectively. A 5 (domain) × 9 (style) × 2 (family structure) MANOVA was conducted to determine whether there were differences in the degree of usage of each of the styles both within and across domains and family structures. Domain and style were within-subjects factors, whereas family structure was a between-subjects factor. There was no main effect for polygamy, $F(1, 86) = 0.58$, $p = 0.450$, nor a significant polygamy × style interaction, $F(8, 79) = 1.59$, $p = 0.140$, nor a significant three-way interaction, $F(32, 55) = 0.68$, $p = 0.875$. Thus, the two groups had an overall similar profile of styles within each domain.

**TABLE 3**
**Percent Responding in Each Group on Question of Who Should Have Right to Decide Matters in Each Domain**

| Domain | Parents | Myself | Both | *p* Value |
|---|---|---|---|---|
| **Appearance** | | | | 0.972 |
| Monogamy | 10.3 | 47.6 | 42.1 | |
| Polygamy | 10.0 | 46.3 | 43.8 | |
| **Career** | | | | 0.893 |
| Monogamy | 10.9 | 47.9 | 41.2 | |
| Polygamy | 13.0 | 48.1 | 39.0 | |
| **Money** | | | | 0.472 |
| Monogamy | 19.2 | 42.4 | 38.4 | |
| Polygamy | 14.1 | 39.7 | 46.2 | |
| **Going out** | | | | 0.600 |
| Monogamy | 10.5 | 49.2 | 40.3 | |
| Polygamy | 13.2 | 42.1 | 44.7 | |
| **Privacy** | | | | 0.806 |
| Monogamy | 16.1 | 43.5 | 40.3 | |
| Polygamy | 12.8 | 46.2 | 41.0 | |

*Note: p* Values result from a Chi-square test with 2 degrees of freedom within each domain.

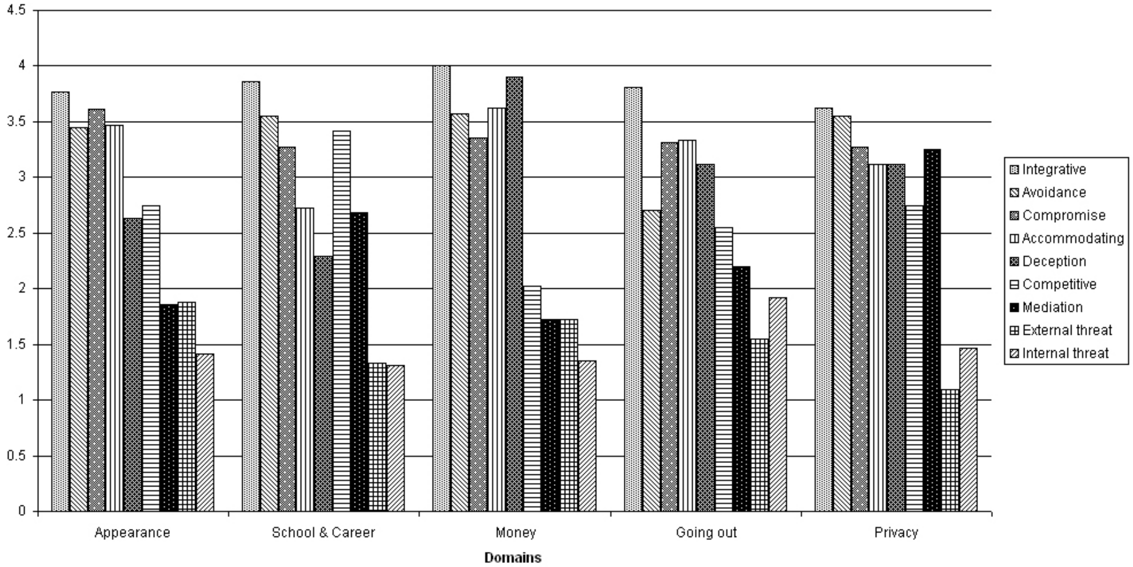

**FIGURE 1**. Conflict Styles in Each Domain in Monogamous Families

### DISCUSSION

The last 20 years have seen a growing body of research dealing with the institution of polygamy and its effects on children[16]. Most of this research, however, has examined only the family

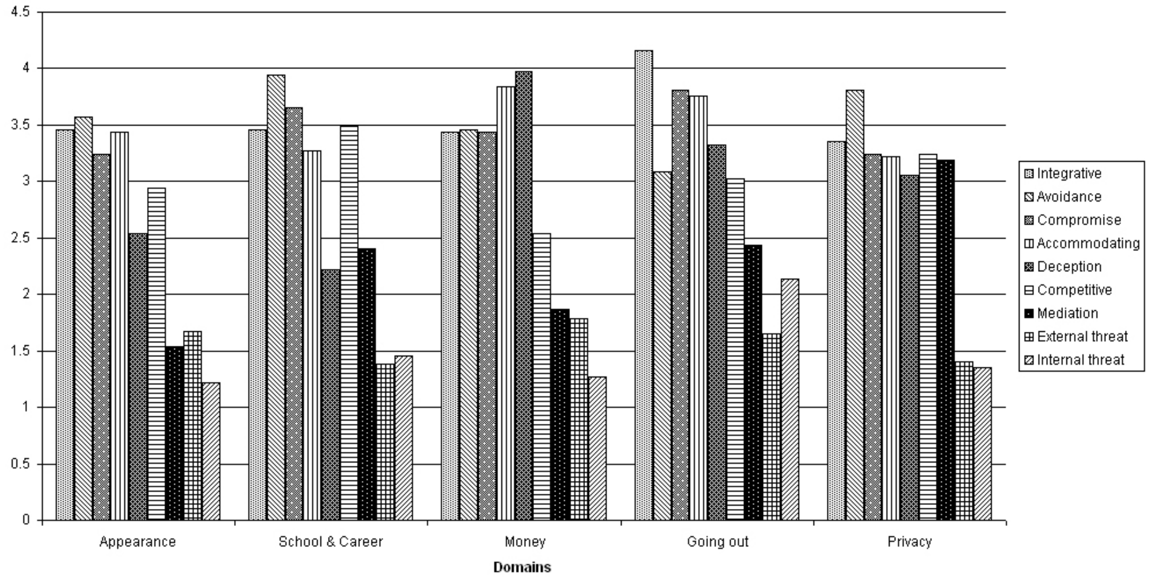

**FIGURE 2.** Conflict Styles in Each Domain in Polygamous Families structure (polygamous or monogamous) as an explanatory variable for the effects on children, and this limited approach has led to conflicting findings. For example, in one Bedouin-Arab community, some studies have suggested that the high school students from polygamous families are doing as well as their peers in monogamous families[1,32], while other studies have identified disadvantages for those same children[11]. The present authors assert that much of this uncertainty can be attributed to excessive reliance on a one-factor conceptual framework based on family structure. Family structure alone is inadequate for explaining the effects of polygamy on children. There is a need for further research that will evaluate the effects of mediating and moderating factors within the family (such as intrafamily conflict). According to Elbedour et al.[16], careful attention must be given to the task of identifying and examining the mediating and moderating variables that to some extent determine child development outcomes in polygamous families.

The current study of children in the Arab community assesses one of these mediating processes — conflict between parents and children. In designing this study, we expected that polygamous family structure would be associated with a greater frequency of parent-child conflict. This expectation was drawn from previous studies that have established a correlation between polygamy and other forms of intrafamily conflict, such as competition between wives[19], rivalry between full- and half-siblings[15], and unequal treatment of wives by the husband[20]. In other words, we expected that the high potential for parental conflict in polygamous families and the exposure of children to this marital stress would increase the likelihood of parent-child conflict and affect the way the children would respond to conflict. Compared with their peers from monogamous families, we expected that children from monogamous families would tend to use different styles of conflict resolution and be more likely to use violent or aggressive means for resolving conflicts.

However, the results of the study do not support either of these hypotheses. Across the five domains that we examined (privacy, career, money, appearance, and leisure pursuits), the frequency of parent-child conflict in polygamous families was not significantly different than those reported by subjects in monogamous families in the same community. The results showed no differences in the use of various conflict management styles across domains and family structures. Overall, within each domain, the two groups (children from monogamous and polygamous families) had a similar profile of conflict resolution styles.

These results raise a number of unresolved issues that require further hypothesizing. One possible explanation is that the children in polygamous families distance themselves from their parents' quarrels. A second possibility is that the marital conflicts reported by previous investigators were based on mere assertion. Another potential explanation is that the conflicts within these families are not serious enough to spill over into the parent-child relationship.

A third explanation is that a supportive cultural context (e.g., collectivity and extended families within the Bedouin-Arab community) can moderate the stressful effects of a polygamous family structure. Cultural context affects the etiology and development of maladaptive behavior[39] as well as the behavior that is viewed as normal and desirable in the culture[40]. Since polygamy is common in this community, and no cultural stigmas are associated with it, these cultural values may promote resilience or act as a positive moderator of parent-child conflict within polygamous families.

### CONCLUSIONS

Overall, the results of the current study demonstrate that polygamy is a complex phenomenon. Although the results of this study do not show a conclusive association between polygamy and parent-child conflict, they also do not eliminate the possibility that such a link exists. The lack of exacerbated parent-child conflict in the five domains examined in this study does not necessarily mean that polygamous families do not have a higher rate of these conflicts. The present study relies heavily on self-reported single informants (the adolescents themselves) and a single assessment method. Finally, it is possible that the domains that we used were not sensitive or specific enough to capture the nature, intensity, or duration of the conflict in these families.

### BIOSKETCHES

**Salman Elbedour**, **Ph.D.,** is an Associate Professor in the Department of Human Development and Psychoeducational Studies, School of Education, Howard University, Washington, D.C. He earned his Ph.D. in Educational/School Psychology and two of his master's degrees at the University of Minnesota. His research topics primarily involve disadvantaged and under-served populations such as Bedouin-Arab minority, and other topics that relate to developmental psychopathology, political violence, psychological traumatization, child abuse, and neglect. Currently he is the coordinator of the Urban School Psychology at Howard University. E-mail: selbedour@howard.edu

**Joel M. Hektner**, **Ph.D.,** is an Assistant Professor in Child Development and Family Science in the Department of Child Development and Family Science, North Dakota State University, Fargo. He earned his Ph.D. in Human Development at the University of Chicago. His research topics primarily involve family and peer factors that promote optimal development and evaluations of preventive interventions for antisocial behavior. E-mail: Joel.Hektner@ndsu.nodak.edu

**Mohammed Morad, M.D.,** is Specialist and Lecturer in family medicine at the National Institute of Child Health and Human Development, Division of Community Health, Ben Gurion University of the Negev and the Medical Director of a large area clinic in the city of Beer-Sheva. Dr. Morad has publications on Bedouin health, health aspects, spiritual health and aging in persons with intellectual disability, and is a presenter on topics like health policy and services for disadvantaged at national and international conferences. E-mail: morad62@barak-online.net

**Soleman H, Abu-Bader**, **Ph.D.,** School of Social Work, Howard University, Washington, D.C., received his B.A. in social work from Ben-Gurion University in 1992 and his M.S.W. from Augsburg College in Minneapolis, MN in 1994. In 1998, Dr. Abu-Bader completed his Ph.D. at the University of Utah School of Social Work, Salt Lake City. His dissertation was entitled "Predictors of Work Satisfaction between Arab and Jewish Social Workers in Israel". Dr. Abu-Bader joined the School of Social Work Faculty in Fall 2000. He has written and presented research papers on work satisfaction and burnout among social workers, depression, life satisfaction, and self-esteem of frail elderly, and depression among former welfare recipients. E-mail: sabu-bader@howard.edu

## APPENDIX

*<u>Parent-Child Conflict Scale</u>, Developed by Elbedour, Salman, Ph.D, Department of Human Development and Psychoeducational Studies, School of Education, Howard University, 2441 Fourth Street, NW, Washington, DC 20059, United States of America*

**MONEY:** Please indicate one issue related to money that provokes the most frequent conflicts/arguments between you and your parents.

Please specify_______________________________________________

How often do you have conflicts/arguments with your parents about the money issue you specified?

| | | |
|---|---|---|
| a) Every week | b) Nearly every week | c) About three times a month |
| d) About twice a month | e) About once a month | f) About every six weeks |
| g) About every three months | h) About once or twice a year | i) Less than once a year |
| j) Never | | |

How much influence does each of the following individuals have on the way this conflict/argument is usually resolved?

| | **"No Influence"--------------- ---------------"Large influence"** | | | | | |
|---|---|---|---|---|---|---|
| Father | 0 | 1 | 2 | 3 | 4 | 5 |
| Mother | 0 | 1 | 2 | 3 | 4 | 5 |
| Subject | 0 | 1 | 2 | 3 | 4 | 5 |

*When you have* conflicts/arguments *with your parents about the money issue you specified:*

| | **Definitely not true of me ---Clearly true in my case** | | | | |
|---|---|---|---|---|---|
| 1) I collaborate with my parents to come up with decisions acceptable to us. | **1** | **2** | **3** | **4** | **5** |
| 2) I usually accommodate the wishes of my parents. | **1** | **2** | **3** | **4** | **5** |
| 3) I use "give and take" so that a compromise can be made. | **1** | **2** | **3** | **4** | **5** |
| 4) I try to stay away from disagreements with my parents. | **1** | **2** | **3** | **4** | **5** |
| 5) I do not feel bound to follow the decisions of my parents. | **1** | **2** | **3** | **4** | **5** |
| 6) Different persons acts as mediators to resolve the conflict | **1** | **2** | **3** | **4** | **5** |
| 7) I take advantage of my parent's good moods and then bring up my demands. | **1** | **2** | **3** | **4** | **5** |
| 8) I scream and holler at my parents to convince them to give in to my demands. | **1** | **2** | **3** | **4** | **5** |
| 9) I threaten that if they don't give in to my demands, I will hurt myself. | **1** | **2** | **3** | **4** | **5** |

Do children have the right to decide how to spend their pocket money? YES NO

Who should have the right to decide how to spend your pocket money? Parents Myself Both

In your opinion, who usually wins concerning the money argument you specified?

| 1 | 2 | 3 | 4 | 5 | 6 |
|---|---|---|---|---|---|
| I win all the time | I win most of the time | More often than not | Occasionally | Rarely | Never |

On the whole, how satisfied are you with the way in which conflicts/arguments concerning money between you and your parents end?

| 0 | 1 | 2 | 3 | 4 | 5 | 6 |
|---|---|---|---|---|---|---|
| Extremely Unsatisfied | Fairly Unsatisfied | A little Unsatisfied | Satisfied | Very Satisfied | Extremely Satisfied | Perfectly Satisfied |

**PHYSICAL APPEARANCE:** Please circle only one issue concerning conventionality that provokes the most frequent conflicts/arguments between you and your parents.

1) Clothing 2) Hairstyle 3) Having body piercing 4) Tattoos 5) Other (specify)__________

How often do you have conflicts/arguments with your parents regarding the issue you specified?

a) Every week b) Nearly every week c) About three times a month d) About twice a month e) About once a month f) About every six weeks g) About every three months h) About once or twice a year i) Less than once a year j) Never

How much influence does each of the following individual have in the decision making process concerning the Physical appearance issue you specified?

| | **"No Influence"--------------- ---------------Large influence"** | | | | | |
|---|---|---|---|---|---|---|
| Father | 0 | 1 | 2 | 3 | 4 | 5 |
| Mother | 0 | 1 | 2 | 3 | 4 | 5 |
| Subject | 0 | 1 | 2 | 3 | 4 | 5 |

*When you have* conflicts/arguments *with your parents regarding conventionality:*

| | **Definitely not true of me ---------Clearly true in my case** | | | | |
|---|---|---|---|---|---|
| 1) I exchange accurate information to my parents to solve a problem together. | **1** | **2** | **3** | **4** | **5** |
| 2) I give in to the wishes of my parents | **1** | **2** | **3** | **4** | **5** |
| 3) I try to find a middle course to resolve an impasse. | **1** | **2** | **3** | **4** | **5** |
| 4) I avoid an encounter or confrontation with my parents. | **1** | **2** | **3** | **4** | **5** |
| 5) I am the only one to determine what to wear or dress. | **1** | **2** | **3** | **4** | **5** |
| 6) I seek help from others in order to resolve the argument. | **1** | **2** | **3** | **4** | **5** |
| 7) At the beginning I bring up extreme demands and then compromise on what I really want. | **1** | **2** | **3** | **4** | **5** |
| 8) I show anger and hostility and absolutely threaten them unless they respond to my demands. | **1** | **2** | **3** | **4** | **5** |

9) I threaten to damage my own belongings as a way of pressuring my parents. **1 2 3 4 5**

Should children have the right to decide their own way what to wear or dress? YES NO

Who should have the right to decide for you what to wear or dress? Parents Myself Both

In your opinion, who usually wins the arguments on this topic?

| 1 | 2 | 3 | 4 | 5 | 6 |
|---|---|---|---|---|---|
| I win all the time | I win most of the time | More often than not | Occasionally | Rarely | Never |

On the whole, how satisfied are you with the way in which conflicts/arguments between you and your parents end?

| 0 | 1 | 2 | 3 | 4 | 5 | 6 |
|---|---|---|---|---|---|---|
| Extremely Unsatisfied | Fairly Unsatisfied | A little Unsatisfied | Satisfied | Very Satisfied | Extremely Satisfied | Perfectly Satisfied |

**PRIVACY:** Please circle only one private issue that provokes the most frequent conflicts/arguments between you and your parents.

1) Getting married 2) Choosing girl/boyfriend 3) Other (specify)_________________________

How often do you have conflicts/arguments with your parents regarding the private affairs you specified?

| | | |
|---|---|---|
| a) Every week | b) Nearly every week | c) About three times a month |
| d) About twice a month | e) About once a month | f) About every six weeks |
| g) About every three months | h) About once or twice a year | i) Less than once a year |
| j) Never | | |

How much influence does each of the following individuals have in the decision making process concerning the privacy issue you specified?

| | **"No Influence"--------------- ---------------Large influence"** | | | | | |
|---|---|---|---|---|---|---|
| Father | 0 | 1 | 2 | 3 | 4 | 5 |
| Mother | 0 | 1 | 2 | 3 | 4 | 5 |
| Subject | 0 | 1 | 2 | 3 | 4 | 5 |

*When you have* conflicts/arguments *with your parents regarding your private affairs*:

| | **Definitely not true of me -------Clearly true in my case** | | | | |
|---|---|---|---|---|---|
| 1) I try to investigate an issue with my parents to find a solution acceptable to us. | **1** | **2** | **3** | **4** | **5** |
| 2) I often go along with the suggestions of my parents. | **1** | **2** | **3** | **4** | **5** |
| 3) I negotiate with my parents so that a compromise can be reached. | **1** | **2** | **3** | **4** | **5** |
| 4) I try to avoid unpleasant exchanges with my parents. | **1** | **2** | **3** | **4** | **5** |
| 5) I am generally firm in arguing my side. | **1** | **2** | **3** | **4** | **5** |
| 6) I listen to the opinions and suggestions of others to find a solution. | **1** | **2** | **3** | **4** | **5** |
| 7) I take advantage of situations in which one of my parents is on my side, to convince the other side. | **1** | **2** | **3** | **4** | **5** |
| 8) I threaten my parents that if they do not comply with my wishes, I will tell their secrets to others | **1** | **2** | **3** | **4** | **5** |
| 9) Sometimes I threaten to refuse to eat or drink until they agree to my demands. | **1** | **2** | **3** | **4** | **5** |

Do parents have the right to interfere in their children's privacy? YES NO

Who should have the right to interfere with your privacy? Parents Myself Both

In your opinion, who usually wins the arguments on this topic?

| 1 | 2 | 3 | 4 | 5 | 6 |
|---|---|---|---|---|---|
| I win all the time | I win most of the time | More often than not | Occasionally | Rarely | Never |

On the whole how satisfied are you with the way in which conflicts/arguments between you and your parents end?

| 0 | 1 | 2 | 3 | 4 | 5 | 6 |
|---|---|---|---|---|---|---|
| Extremely Unsatisfied | Fairly Un satisfied | A little Unsatisfied | Satisfied | Very Satisfied | Extremely Satisfied | Perfectly Satisfied |

**GOING OUT:** Leisure time interests and activities. Please circle only one issue concerning leisure time interests and activities that provoke the most frequent conflicts/arguments between you and your parents.

1) Where and when to go out 2) who to go out with 3) Other (specify)__________________________________

How often do you have conflicts/arguments with your parents regarding the issue you specified?

| | | |
|---|---|---|
| a) Every week | b) Nearly every week | c) About three times a month |
| d) About twice a month | e) About once a month | f) About every six weeks |
| g) About every three months | h) About once or twice a year | i) Less than once a year |
| j) Never | | |

How much influence does each of the following individuals have in the decision making process concerning the leisure issue you specified?

**"No Influence"--------------- ---------------Large influence"**

| | | | | | | |
|---|---|---|---|---|---|---|
| Father | 0 | 1 | 2 | 3 | 4 | 5 |
| Mother | 0 | 1 | 2 | 3 | 4 | 5 |
| Subject | 0 | 1 | 2 | 3 | 4 | 5 |

*When you have* conflicts/arguments *with your parents regarding the issue you specified:*

| | **Definitely not true of me -------Clearly true in my case** | | | | |
|---|---|---|---|---|---|
| 1) I try to integrate or blend my ideas with those of my parents to come up with a decision together. | **1** | **2** | **3** | **4** | **5** |
| 2) I often go along with the suggestions of my parents | **1** | **2** | **3** | **4** | **5** |
| 3) I use "give and take" so that a compromise can be made. | **1** | **2** | **3** | **4** | **5** |
| 4) I usually avoid open discussion of my differences with my parents. | **1** | **2** | **3** | **4** | **5** |
| 5) When conflict arise, I follow my own mind and ideas. | **1** | **2** | **3** | **4** | **5** |
| 6) I invite a third party to intervene in the conflict by providing, information, making suggestions and reframing the issue. | **1** | **2** | **3** | **4** | **5** |
| 7) I compare my situation to my peers who ask their parents to satisfy their needs like other parents do | **1** | **2** | **3** | **4** | **5** |
| 8) I generally use anger, swearing to let my parents pay attention to my side. | **1** | **2** | **3** | **4** | **5** |
| 9) I isolate and cry bitterly or sleep beyond what's acceptable in order to attract my parents' attention, with the purpose of acquiring what I want. | **1** | **2** | **3** | **4** | **5** |

Do children have the right to decide on his/her leisure time? YES NO

Who should have the right to decide your leisure time interests and activities? Parents Myself Both

In your opinion, who usually wins the arguments on this topic?

| 1 | 2 | 3 | 4 | 5 | 6 |
|---|---|---|---|---|---|
| I win all the time | I win most of the time | More often than not | Occasionally | Rarely | Never |

On the whole, how satisfied are you with the way in which conflicts/arguments between you and your parents end?

| 0 | 1 | 2 | 3 | 4 | 5 | 6 |
|---|---|---|---|---|---|---|
| Extremely Unsatisfied | Fairly Unsatisfied | A little Unsatisfied | Satisfied | Very Satisfied | Extremely Satisfied | Perfectly Satisfied |

**SCHOOLING &CAREER DECISIONS:** Please circle only one career decision issues that provokes the most frequent conflicts/arguments between you and your parents.

"Please indicate one career decision issue that provokes the most frequent conflicts/arguments between you and your parents.

Please specify: ------------------------------------------------"

How often do you have conflicts/arguments with your parents regarding your career decisions?

a) Every week b) Nearly every week c) About three times a month d) About twice a month e) About once a month f) About every six weeks g) About every three months h) About once or twice a year i) Less than once a year i j) Never

How much influence does each of the following individual have in the decision making process concerning the career issue you specified?

| | **"No Influence"--------------- ---------------Large influence"** | | | | | |
|---|---|---|---|---|---|---|
| Father | 0 | 1 | 2 | 3 | 4 | 5 |
| Mother | 0 | 1 | 2 | 3 | 4 | 5 |
| Subject | 0 | 1 | 2 | 3 | 4 | 5 |

*When you have* conflicts/arguments *with your parents regarding career decisions:*

| | **Definitely not true of me ------Clearly true in my case** | | | | |
|---|---|---|---|---|---|
| 1) I try to work with my parents to find solutions to a problem which satisfy both our expectations. | **1** | **2** | **3** | **4** | **5** |
| 2) I usually allow concessions to my parents. | **1** | **2** | **3** | **4** | **5** |
| 3) I try to find a middle course to resolve an impasse. | **1** | **2** | **3** | **4** | **5** |
| 4) I try to stay away from disagreements with my parents. | **1** | **2** | **3** | **4** | **5** |
| 5) I am always self reliant and independent in making my decisions. | **1** | **2** | **3** | **4** | **5** |
| 6) I try to talk to a brother or sister or someone to convince my parents. | **1** | **2** | **3** | **4** | **5** |
| 7) I stay mad, and get madder the more I talk on the topic is one way to cheat my parents and persuade them | **1** | **2** | **3** | **4** | **5** |
| 8) I use violence or threats of violence toward my parents, brothers or sisters, in order to get anything. | **1** | **2** | **3** | **4** | **5** |
| 9) I threaten to damage my own belongings as a way of pressuring my parents. | **1** | **2** | **3** | **4** | **5** |

Do children have the right to decide their future career interests and activities? YES NO

Who should have the right to decide your future career interests and activities? Parents Myself Both

In your opinion, who usually wins the arguments on the topic you specified?

| 1 | 2 | 3 | 4 | 5 | 6 |
|---|---|---|---|---|---|
| I win all the time | I win most of the time | More often than not | Occasionally | Rarely | Never |

On the whole, how satisfied are you with the way in which conflicts/arguments between you and your parents end?

| 0 | 1 | 2 | 3 | 4 | 5 | 6 |
|---|---|---|---|---|---|---|
| Extremely Unsatisfied | Fairly Unsatisfied | A little Unsatisfied | Satisfied | Very Satisfied | Extremely Satisfied | Perfectly Satisfied |
